# Acute Effects of Red Chili, a Natural Capsaicin Receptor Agonist, on Gastric Accommodation and Upper Gastrointestinal Symptoms in Healthy Volunteers and Gastroesophageal Reflux Disease Patients

**DOI:** 10.3390/nu12123740

**Published:** 2020-12-04

**Authors:** Tanisa Patcharatrakul, Chatchai Kriengkirakul, Tawatchai Chaiwatanarat, Sutep Gonlachanvit

**Affiliations:** 1 Center of Excellence on Neurogastroenterology and Motility, Department of Medicine, Faculty of Medicine, Chulalongkorn University, Bangkok 10330, Thailand; dr_tanisa@yahoo.com (T.P.); chatchaikrieng@yahoo.com (C.K.); 2 Division of Gastroenterology, Department of Medicine, King Chulalongkorn Memorial Hospital, The Thai Red Cross Society, Bangkok 10330, Thailand; 3 Division of Nuclear Medicine, Department of Radiology, Faculty of Medicine, Chulalongkorn University, Bangkok 10330, Thailand; tawatchai.ch@chula.ac.th

**Keywords:** chili, capsaicin, GERD, gastric accommodation, stomach

## Abstract

The effects of chili on gastric accommodation (GA) in gastroesophageal reflux disease (GERD) patients have not been explored. Methods: In total, 15 healthy volunteers (HV) and 15 pH-positive non-erosive GERD (NERD) patients underwent single-photon emission computed tomography after ingesting 2 g of chili or placebo in capsules in a randomized double-blind crossover fashion with a one-week washout period. GA was the maximal postprandial gastric volume (GV) after 250 mL of Ensure^®^ minus the fasting GV. Upper gastrointestinal symptoms were evaluated by using a visual analog scale. Results: NERD patients but not HV had significantly greater GA after chili compared to a placebo (451 ± 89 vs. 375 ± 81 mL, *p* < 0.05). After chili, the postprandial GVs at 10, 20, and 30 min in NERD patients were significantly greater than HV (10 min, 600 ± 73 vs. 526 ± 70 mL; 20 min, 576 ± 81 vs. 492 ± 78 mL; 30 min, 532 ± 81 vs. 466 ± 86 mL, all *p* < 0.05). In NERD, chili was associated with significantly less satiety, more severe abdominal burning (*p* < 0.05), and a trend of more severe heartburn (*p* = 0.06) compared to the placebo. In HV, postprandial symptoms after chili and placebo ingestion were similar (*p >* 0.05). Conclusions: Chili enhanced GA in NERD patients but not in HV. This suggests that the modulation of GA in NERD is abnormal and likely involves transient receptor potential vanilloid 1 (TRPV1) sensitive pathways.

## 1. Introduction

Capsaicin (8-methyl-N-vanillyl-6-nonenamide) is a major component of chili, which is a common food ingredient worldwide. It can stimulate the transient receptor potential vanilloid 1 (TRPV1) receptors in the gastrointestinal tract and can produce pain and a burning sensation in humans [1,2,3]. Abdominal burning, abdominal pain, and heartburn often develop after chili ingestion in patients with peptic ulcer disease, functional dyspepsia, and gastroesophageal reflux disease (GERD) [4,5], suggesting the role of capsaicin-sensitive nociceptive pathways in the development of symptoms in these patients. Although the effect of chili on gastrointestinal sensation has been well demonstrated, the effect of chili on gastric motor function has not been well explored. Previous studies demonstrated that ingestion of a meal with capsaicin in a capsule delay gastric emptying [5,6,7,8] but increase small bowel transit time in humans [6,7]. Acute administration of red pepper containing capsaicin in the stomach has been reported to decrease proximal gastric tone, inhibit phasic contractility of the proximal stomach, and increase sensitivity to proximal gastric distension in healthy volunteers [9]. A preliminary study in our laboratory demonstrated that patients with non-erosive reflux disease (NERD) had delayed gastric emptying time during the first hour after having a meal with chili and an increasing number of acid refluxes in the second hour [10]. In addition, patients with NERD have been reported to have an increased number of TRPV 1 receptors or capsaicin receptors in the gastrointestinal tract compared to healthy volunteers [11,12]. Gastric accommodation is the relaxation reflex of the proximal stomach in response to meal ingestion. Enhanced gastric accommodation reflex has been reported in patients with GERD [13] and is associated with an increased rate of transient lower esophageal sphincter relaxation [14]. The effect of chili containing capsaicin on gastric accommodation in humans has not been explored. This study aimed to investigate the effects of red chili on gastric accommodation and upper gastrointestinal symptoms in NERD patients compared to healthy volunteers. Through this study, we hope to gain insight into the role of capsaicin mediated neuronal pathways in the modulation of gastric accommodation in GERD patients and healthy humans.

## 2. Materials and Methods

### 2.1. Subjects

Healthy volunteers and patients with NERD, between the ages of 18 and 65, were enrolled at King Chulalongkorn Memorial Hospital, Bangkok, Thailand. The healthy volunteers had no history of gastrointestinal disease and intra-abdominal surgery, except for appendectomies, no gastrointestinal symptoms, and had not taken any medication during the past month. The inclusion criteria for NERD patients were as follows: (i) they had either heartburn or regurgitation as their main symptom, had no esophagitis, and had a normal stomach evaluated by an upper gastrointestinal endoscopy during the past 12 months; and (ii) they had a positive 24-h esophageal pH monitoring result as defined by the total percent time of pH < 4 at distal esophagus more than 4.5%. The patients were excluded if they had diabetes mellitus, hypertension, a neurologic disease, were pregnant, or were on medication(s), including proton pump inhibitors, H2-receptor antagonists, antispasmodics, prokinetics, antidepressants, medications with anticholinergic effects, and other medications with gastrointestinal motility/sensation effects. Patients were unable to stop taking them at least seven days before the study. All subjects gave their informed consent for inclusion before they participated in the study. The study was conducted in accordance with the Declaration of Helsinki, and the protocol was approved by the Ethics Committee of the Faculty of Medicine, Chulalongkorn University, Thailand (IRB number 067/52).

### 2.2. Methods

**Figure 1:**
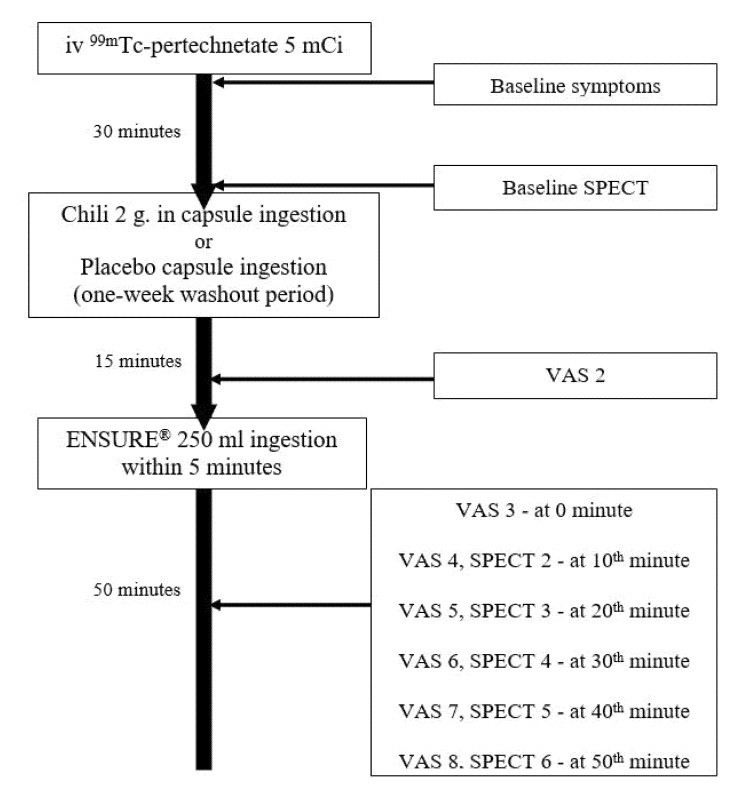
A schematic outline of the study protocol. SPECT: single-photon emission computed tomography; VAS: visual analog scale.

Each participant underwent a single-photon emission computed tomography (SPECT) for a gastric accommodation study after ingestion of 2 g of red chili (Capsicum frutescens Linn, 1.46 mg capsaicin per 2 g dry weight, Chili Powder, Artchit International Pepper and Spice, Co Ltd., Bangkok, Thailand) or placebo in capsules in a randomized double-blind crossover fashion with a one-week washout period. All subjects reported to the gastrointestinal laboratory at the Center of Excellence on Neurogastroenterology and Motility, Chulalongkorn University, Bangkok, Thailand at 8:00 a.m. after an overnight fast. For each study, the baseline fasting gastric scintigraphy images were acquired at 30 min after intravenous 99mTc-pertechnetate. Then, the chili or placebo was administered and all subjects ingested standard liquid meals (250 mL of Ensure) 15 min later. Postprandial scintigraphic images were acquired immediately and then every 10 min for 50 min. The gastric accommodation volume was the maximal postprandial gastric volume minus the fasting gastric volume. Upper gastrointestinal symptoms (abdominal burning, abdominal pain, heartburn, nausea/vomiting, abdominal fullness, early satiation, food regurgitation, acid regurgitation, and belching) were evaluated using a 10-cm long visual analog scale at baseline 10 min after chili or placebo ingestion and immediately every 10 min after liquid meal ingestion. The gastric accommodation and upper gastrointestinal symptoms were compared between chili and placebo ingestions and compared between patients with NERD and healthy volunteers. The schematic outline of the study protocol is shown in Figure 1.

The gastric accommodation studies were performed in a supine position using SPECT as previously reported from our center after intravenous administration of 185 MBq (5 mCi) 99mTc-pertechnetate (total dose = 4 millisievert per subject from 2 examinations) [15].

In brief, SPECT was performed using a triple-head gamma camera (Triad XLT 20, Trionix, Twinsburg, OH, USA) equipped with low-energy ultrahigh-resolution collimators. Gastric scintigraphic images were acquired into a 128 × 128 matrix, every 5° at 20 s per image, taking 10 min to complete 360°. These data were subsequently reconstructed using filtered back-projection (Ramp–Butterworth filter, order 10, cut-off 0.45 Nyquist) to produce transaxial images of the stomach. The transaxial images of the stomachs were subsequently analyzed using the freely available Image J software (National Institute of Health, Bethesda, MD, USA). Finally, the gastric volume was measured.

### 2.3. Statistical Analysis

The sample size was calculated based on the data available from a previous study of gastric accommodation in healthy volunteers [15]. At least 15 subjects were needed for this cross-over study to determine a 30% difference between gastric accommodation value after chili ingestion in healthy volunteers or patients with NERD and the normal gastric accommodation value after placebo ingestion in healthy volunteers with 90% power at α = 0.05. The data were analyzed using SPSS software for Windows (version 22.0, SPSS Inc., Chicago, IL, USA). A paired or unpaired student *t*-test, Mann–Whitney *u* test, or Wilcoxson sign rank test was used as appropriate to determine a statistically significant difference when comparing chili and the placebo or patients with NERD and healthy volunteers. A *p*-value of <0.05 was considered statistically significant.

## 3. Results

In total, 15 healthy volunteers (age 40 ± 13 years, 8 females) and 15 patients with NERD (age 48 ± 9 years, 8 females) completed the studies. The gender, age, BMI, and baseline gastric volume were not significantly different between NERD patients and healthy volunteers (Table 1). No volunteers reported regular alcohol consumption. NERD patients had higher baseline symptom scores for abdominal burning, abdominal pain, heartburn, abdominal fullness, food regurgitation, and belching than healthy volunteers (*p* < 0.05) (Table 1).

### 3.1. The Gastric Accommodation after Chili and Placebo Ingestion

**Figure 2:**
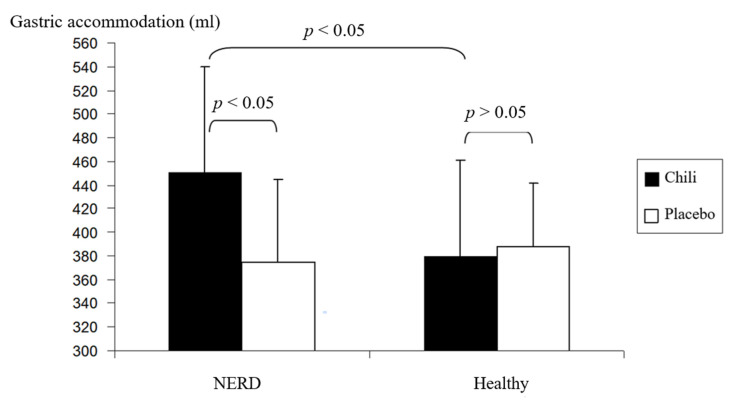
The gastric accommodation after chili and placebo ingestion in healthy volunteers and patients with NERD.

Gastric accommodation after chili ingestion was significantly increased compared to placebo ingestion in NERD patients (451 ± 89 vs. 375 ± 81 mL, *p* < 0.05). Gastric accommodation in healthy volunteers was not significantly different between chili and placebo ingestion (380 ± 69 vs. 388 ± 54 mL, *p >* 0.05) (Figure 2). Furthermore, gastric accommodation of NERD patients was significantly increased compared to healthy volunteers after chili ingestion (*p* < 0.05) (Figure 2). Gastric accommodation after placebo ingestion was not significantly different between NERD patients and healthy volunteers (*p >* 0.05) (Figure 2).

### 3.2. The Gastric Volume after Chili and Placebo Ingestion

**Figure 3:**
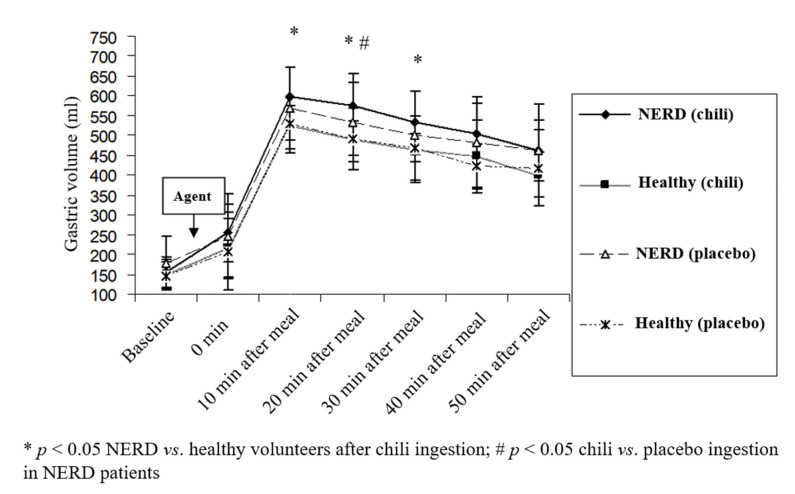
The effect of chili and the placebo on postprandial gastric volume in healthy volunteers and patients with NERD.

In healthy volunteers, the postprandial gastric volume after chili ingestion at each time point was not significantly different from that of placebo ingestion (*p >* 0.05). In contrast, the postprandial gastric volume at 20 min after chili ingestion in NERD patients was significantly greater than placebo ingestion (*p* < 0.05) (Figure 3). After chili ingestion, the gastric volumes at 10, 20, and 30 min postprandial period in NERD patients were significantly greater than healthy volunteers (600 ± 73 vs. 526 ± 70, 576 ± 81 vs. 492 ± 78, 532 ± 81 vs. 466 ± 86 mL, NERD vs. healthy volunteers at a postprandial period of 10, 20, and 30 min, respectively, *p* < 0.05) (Figure 3). Although the postprandial gastric volume after placebo ingestion in NERD patients was greater than healthy volunteers, the difference did not reach statistical significance (*p >* 0.05).

### 3.3. Postprandial Upper Gastrointestinal Symptom Scores after Chili and Placebo Ingestion

**Figure 4:**
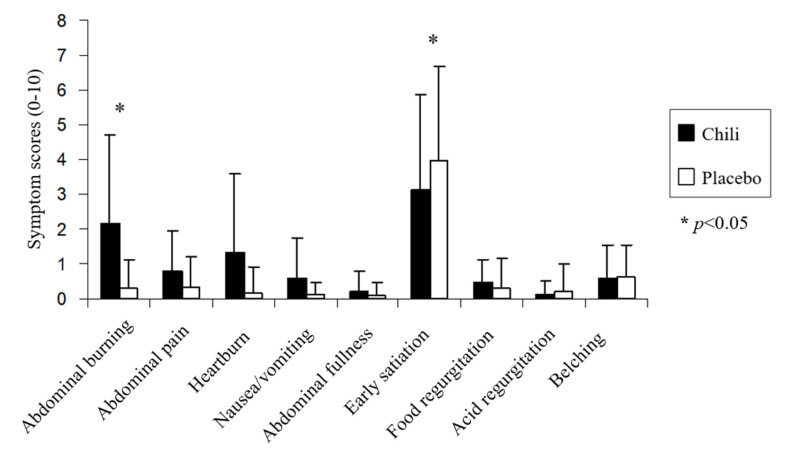
Upper gastrointestinal symptoms scores after chili or placebo ingestion in patients with non-erosive gastroesophageal reflux disease.

Chili ingestion was associated with significantly higher postprandial abdominal burning symptom severity compared to the placebo in NERD patients (2.2 ± 2.5 vs. 0.3 ± 0.8; *p* < 0.05) (Figure 4). The early satiety symptom severity in NERD patients after chili ingestion was significantly less severe compared to the placebo (3.1 ± 2.7 vs. 3.9 ± 2.7; *p* < 0.05). The typical reflux and other upper GI symptoms were not significantly different between chili and the placebo in NERD patients (*p >* 0.05); whereas, the heartburn severity tended to be higher after chili compared to placebo ingestion.

**Figure 5:**
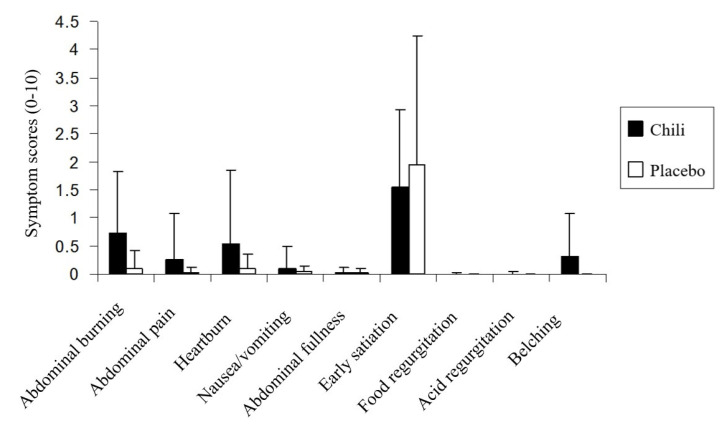
Upper gastrointestinal symptoms scores after chili and placebo ingestion in healthy volunteers.

In contrast, each of the postprandial upper GI symptoms (abdominal burning, abdominal pain, heartburn, nausea/vomiting, abdominal fullness, early satiation, food regurgitation, acid regurgitation, and belching) after chili and placebo ingestion were not significantly different (*p >* 0.05) in healthy volunteers (Figure 5).

## 4. Discussion

This is the first study to investigate the gastric accommodation in response to capsaicin containing chili and placebo ingestion in NERD patients compared to healthy volunteers. The gastric volume was measured using SPECT, which was validated in our laboratory [15]. We found that the ingestion of chili, a natural capsaicin receptor agonist, induced greater gastric accommodation volume compared to the placebo in NERD patients, but this effect was not found in healthy volunteers. The finding is new and demonstrates that abnormal gastric accommodation in response to specific foods is present in NERD. Our results suggest that TRPV1 pathways are involved in the postprandial gastric accommodation abnormality in NERD patients. Acid, heat, alcohol, and capsaicin have been known to activate TRPV1 receptors. Thus, it is conceivable that gastric accommodation in response to acidic foods, high-temperature foods (heat), other capsaicin- containing foods, and alcohol may also be abnormal, such as the response to chili in NERD patients.

Previous studies using the barostat technique demonstrated that gastric accommodation in GERD patients was greater than that of healthy volunteers [16,17]. In addition, a positive correlation between the degree of gastric accommodation and the number of transient lower esophageal sphincter relaxations (TLESRs) in GERD patients has been reported [13]. However, another study using intragastric pressure measurement by manometry suggested a negative correlation between gastric accommodation and the rate of TLESRs [18]. These conflicting results may be caused by the different techniques used for the study of gastric accommodation.

Capsaicin is the major ingredient of chili and the TRPV1 receptors have been found to increase in the upper gut of NERD patients [11]. Whether or not the increase in TRPV1 receptors in the upper gut in NERD patients plays a role in the findings of our study is still unknown. In a previous study, Lee KJ et al. reported that the infusion of chili sauce (capsaicin 0.84 mg) into the stomach via a nasogastric tube in healthy volunteers can decrease the gastric tone of the proximal stomach, measured using the barostat technique, with no significant effect on gastric accommodation to a meal, which is similar to our findings in healthy volunteers [9].

The finding that chili could induce abdominal burning symptoms in NERD patients but not in healthy volunteers suggests that the upper gut of a NERD patient is hypersensitive to chili. Previous studies have shown that chili or capsaicin can induce abdominal burning, abdominal pain, and heartburn via c-fiber and TRPV1 receptors [19,20,21] and that chili ingestion can aggravate these symptoms in GERD patients [4,5]. Moreover, our study also has shown a significant decrease in early satiety symptom scores in NERD patients after a meal with chili. The decrease of early satiety symptoms by chili in our study may be explained by the effect of chili on postprandial gastric volume changes or gastric accommodation. A previous study demonstrated the association between impaired gastric accommodation and early satiety symptoms [22]. Red chili has been used in remedies for increasing appetite in Thai traditional medicine [23].

In this study, we used red chili instead of pure capsaicin because we intended to demonstrate the effects of a natural capsaicin agonist that people around the world ingest in their daily life on gastric accommodation in NERD patients. A previous study showed that chili provides a broader modification of the whole nerve fiber that subserves the TRPV1 receptor, whereas the capsaicin receptor antagonists inhibit only a specific group of receptors [24]. Thus, the effects on gut symptoms of chili should be broader. Heartburn and acid regurgitation symptoms were not significantly increased after chili ingestion, especially when compared to the placebo in NERD patients in our study, which could have been due to the small sample size.

The limitations of this study were as follows: (1) the small sample size (15 in each group) may have limited the ability of this study to determine the gastroesophageal reflux symptom differences after chili vs. placebo ingestion in NERD patients; and (2) we performed the SPECT in a supine position, which is not the normal postprandial physiologic position. However, these limitations should not affect the gastric accommodation results but may affect gastroesophageal reflux development and symptoms. Although it is unlikely, we cannot exclude the possibility that other components beside capsaicin in the red chili may contribute to the effect of red chili on gastric accommodation. To the best of our knowledge, there was no candidate component in red chili reported to affect gastric accommodation as found in this study.

## 5. Conclusions

In conclusion, chili, a common food ingredient, can modulate both sensation and motor function of the stomach causing more abdominal burning symptoms and increase gastric accommodation in patients with NERD but not in healthy volunteers. Chili can decrease early satiety symptom, which is possible from its effect on gastric accommodation. This study provides an insight into the role of capsaicin pathways on the pathogenesis of enhancing gastric accommodation and gastroesophageal refluxes in NERD patients.

## Figures and Tables

**Table 1:** The characteristics of patients with NERD and healthy volunteers.

Patients’ Characteristics	NERD (*N* = 15)	Healthy (*N* = 15)
Gender (male:female)	7:08	7:08
Age (years)	48 ± 9	40 ± 13
BMI (kg/m^2^)	24.6 ± 4.3	24.3 ± 3.2
Total % time pH < 4	9.7 ± 5.2	-
Baseline gastric volume (ml)	155.0 ± 39.3	148.5 ± 39.8
Symptoms scores (VAS 0-10)		
- Abdominal burning	2.4 ± 2.9 **	0.08 ± 0.32
- Abdominal pain	2.4 ± 2.7 ***	0
- Heartburn	1.5 ± 2.3 *	0.03 ± 0.12
- Nausea/vomiting	0.54 ± 1.81	0.08 ± 0.33
- Abdominal fullness	2.5 ± 2.8 **	0.1 ± 0.4
- Early satiation	0.9 ± 2.1	0.3 ± 0.7
- Food regurgitation	1.4 ± 2.5 *	0
- Acid regurgitation	0.5 ± 1.8	0
- Belching	1.8 ± 2.5 *	0.2 ± 0.9

Data showed as mean ± SD; * *p* < 0.05, ** *p* < 0.01, *** *p* < 0.005 (NERD vs. healthy volunteers). (NERD: Non-erosive gastroesophageal reflux disease; BMI: body mass index; VAS: visual analog scale).
